# Truncated satratoxin gene clusters in selected isolates of the atranone chemotype of *Stachybotrys chartarum* (Ehrenb.) S. Hughes

**DOI:** 10.1007/s12550-019-00371-x

**Published:** 2019-08-21

**Authors:** Sebastian Ulrich, Ludwig Niessen, Julia Ekruth, Cornelius Schäfer, Florian Kaltner, Christoph Gottschalk

**Affiliations:** 1 Chair of Bacteriology and Mycology, Faculty of Veterinary Medicine, Ludwig-Maximilians-University Munich, Veterinärstraße 13, 80539 Munich, Germany; 2 Chair of Technical Microbiology, TUM School of Life Sciences Weihenstephan, Technical University of Munich, Gregor-Mendel-Str. 4, 85354 Freising, Germany; 3 Chair of Food Safety, Faculty of Veterinary Medicine, Ludwig-Maximilians-University Munich, Schoenleutnerstr. 8, 85764 Oberschleissheim, Germany

**Keywords:** *Stachybotrys*, Chemotype, Genotype, Macrocyclic trichothecenes, Triplex PCR

## Abstract

The fungus *Stachybotrys* (*S*.) *chartarum* was isolated from culinary herbs, damp building materials, and improperly stored animal forage. Two distinct chemotypes of the fungus were described that produced either high-cytotoxic macrocyclic trichothecenes (S type) or low-cytotoxic atranones (A type). Recently, two distinct gene clusters were described that were found to be necessary for the biosynthesis of either macrocyclic trichothecenes (21 *SAT* (*Satratoxin*) genes) or atranones (14 *ATR* (*Atranone*) genes). In the current study, PCR primers were designed to detect *SAT* and *ATR* genes in 19 *S. chartarum* chemotype S and eight *S. chartarum* chemotype A strains. Our analysis revealed the existence of three different genotypes: satratoxin-producing strains that harbored all *SAT* genes but lacked the *ATR* gene cluster (genotype S), non-satratoxin-producing strains that possessed the *ATR* genes but lacked *SAT* genes (genotype A), and a hitherto undescribed hybrid genotype among non-satratoxin-producing strains that harbored all *ATR* genes and an incomplete set of *SAT* genes (genotype H). In order to improve the discrimination of genotypes, a triplex PCR assay was developed and applied for the analysis of *S. chartarum* and *S. chlorohalonata* cultures. The results show that genes for macrocyclic trichothecenes and atranones are not mutually exclusive in *S. chartarum*. Correlation of the new genotype-based concept with mycotoxin production data shows also that macrocyclic trichothecenes are exclusively produced by *S. chartarum* genotype S strains.

## Introduction

*Stachybotrys* (*S*.) spp*.* were detected on dead plant materials (e.g., herbs, straw, and hay) and other cellulosic substrates (Biermaier et al. 2015; El-Kady and Moubasher 1982). Due to its high cellulolytic potential, *Stachybotrys* spp*.* were, together with a variety of other fungal genera, demonstrated to grow on building materials (e.g., wallpaper, plasterboard, or wooden lining) under high water activity conditions (Gravesen et al. 1999; Nielsen et al. 2004). A strong association was detected between *Stachybotrys* ssp*.* and gypsum materials as well as wall papers (Andersen et al. 2011). On these materials, *Stachybotrys* spp. were also associated with other fungi, i.e., *Acremonium* spp., *Ulocladium* spp., and *Penicillium chrysogenum*. *S. chartarum* and *S. chlorohalonata* were found to be the most frequently isolated species of the genus *Stachybotrys* (Wang et al. 2015; Lombard et al. 2016). The former species was sub-divided into two distinct chemotypes, the high-cytotoxic chemotype S and the low-cytotoxic chemotype A. Cultures of the two chemotypes were found to produce either macrocyclic trichothecenes (S type) or atranones (A type) (Jarvis et al. 1995; Andersen et al. 2003; Hinkley et al. 2000, 2003).

In the literature, *S. chartarum* was associated with human (Etzel 2003) and animal (Hintikka 1977a, 1977b) diseases (Johanning and Yang 1994; Nikulin et al. 1994). Stachybotryotoxicosis is a condition that was found to occur in horses, cattle, sheep, and chickens after the oral uptake of *Stachybotrys*-contaminated feed (Forgacs et al. 1958; Kriek and Marasas 1983; Schneider et al. 1979; Vertinsky 1940). In humans, exposure to macrocyclic trichothecenes and other metabolites of *S. chartarum*, e.g., stachylysin, was suspected to cause pulmonary hemorrhage in infants and other symptoms related to the sick building syndrome (SBS) (Dearborn et al. 1999, 2002; Etzel et al. 1998; Vesper et al. 2001, Johanning et al. 1999). Even though there is evidence that links this fungus and its metabolites to several types of illnesses, the causative role of *Stachybotrys* spp. in the etiology of the SBS has been controversially discussed in the more recent literature (Miller et al. 2003; CDC 2002).

Satratoxins and other macrocyclic trichothecenes were shown to have been produced during growth of *S. chartarum* on different building materials. Thus, satratoxin H was proposed as a biomarker for the presence of macrocyclic trichothecenes (Aleksic et al. 2016). Such compounds bind irreversibly to the 60S ribosomal subunit of mammalian cells and inhibit protein biosynthesis (Hernandez and Cannon 1982; Rocha et al. 2005; Ueno 1977). They represent the most cytotoxic trichothecenes currently known (Hanelt et al. 1994). Atranones A and C were found to induce significant inflammatory responses in a mouse lung model (Rand et al. 2006). However, their cytotoxic effects were much lower as compared to macrocyclic trichothecenes (Abbas et al. 2002). Production of atranones and macrocyclic trichothecenes was postulated to be mutually exclusive in strains of the respective chemotypes (Andersen et al. 2003). Strains of the A and the S chemotypes could not be differentiated by cultural and morphological features in previous studies (Andersen et al. 2003). Moreover, matrix-assisted laser desorption/ionization time-of-flight mass spectrometry (MALDI-TOF MS) analysis did not allow for an unequivocal distinction of the two chemotypes (Gruenwald et al. 2015; Ulrich et al. 2016). Andersen et al. (2003) demonstrated that macrocyclic trichothecene-producing strains could be differentiated from atranone producers by the presence of a stable C-to-T exchange at nucleotide position 279 in their *TRI5* genes.

Comparison of the genomes of three *S. chartarum* strains representing both chemotypes as well as one strain of *S. chlorohalonata* revealed the presence of common, but also of chemotype-specific, gene clusters. These were attributed to the biosynthesis of satratoxins and atranones, respectively (Semeiks et al. 2014). Two isolates of *S. chartarum* (IBT 7711 and IBT 40293) produced macrocyclic trichothecenes and contained genes that were suggested to be involved in the respective biosynthetic pathway. Genes *SAT1*–*SAT21* were organized in three *SAT* core gene clusters, SC1–SC3 (Semeiks et al. 2014). Moreover, two atranone-producing isolates (IBT 40288 and *S. chlorohalonata* IBT 40285) lacked all *SAT* genes but instead contained a cluster of 14 *ATR* genes that could have been involved in atranone biosynthesis (Semeiks et al. 2014). From their data, these authors concluded that the absence of the *ATR* gene cluster from the sequenced S-type strains provides evidence for the concept of mutual exclusiveness of *SAT* and *ATR* gene clusters in the two chemotypes of *S. chartarum*.

Based on the earlier findings, we aimed to develop a PCR assay to distinguish the two chemotypes of *S. chartarum*. In this context, we designed primers for the detection of each of the genes within the *SAT* and *ATR* clusters in the chemotypes of *S. chartarum*. Here, we report on findings resulting in the identification of a new genotype of *S. chartarum*. This genotype harbors both the complete *ATR* gene cluster and an incomplete set of *SAT* genes. These result in failure of such strains to produce macrocyclic trichothecenes.

## Materials and methods

### Chemicals

LC-MS-grade acetonitrile (ACN) and methanol were purchased from Th. Geyer (Renningen, Germany). Ultrapure water was obtained by purifying water through an UltraClear® TP UV UF TM from Evoqua Water Technologies (Barsbuettel, Germany). Formic acid and ammonium formate used as additives for LC-MS solvents were obtained from Th. Geyer (Renningen, Germany) and Fluka (Steinheim, Germany), respectively. Standards for roridin E and L-2; satratoxins F, G, and H; and verrucarin J were not commercially available, and these toxins were therefore qualitatively determined after purification of these substances from rice cultures as described earlier (Gareis and Gottschalk 2014).

### Fungal cultures and culture conditions

In this study, 28 fungal strains were used (Table 1). The set of strains comprised six reference strains [available at CBS (Westerdijk Fungal Biodiversity Institute), ATCC (American Type Culture Collection), and IBT Culture Collection of Fungi, Denmark] and 22 field isolates (Culture Collection of Food Safety, Faculty of Veterinary Medicine, Ludwig-Maximilians-University, Munich, Germany, and Chair of Technical Microbiology, TUM School of Life Sciences Weihenstephan, Technical University of Munich, Freising, Germany, from which cultures can be retrieved upon request). The strains were initially characterized using previously described molecular and mass spectrometric methods (Andersen et al. 2003; Ulrich et al. 2016) and were confirmed as either *S. chartarum* or *S. chlorohalonata*. Fungal stock cultures were maintained in glycerol at − 80 °C as described by Niessen et al. (Niessen and Vogel 2010). Working cultures of *Stachybotrys* spp. were grown on 2% malt extract agar plates [MEA, 20 g/L malt extract, 2 g/L soy peptone, and 15 g/L agar (Difco, Heidelberg, Germany), adjust to pH 5.4]. Prior to use, all media were sterilized by autoclaving at 121 °C for 15 min. All cultures were grown at ambient temperature (AT, 22 ± 1 °C). For DNA extraction, cultures were grown in 50 mL of malt extract broth in 100-mL Erlenmeyer flasks on a horizontal shaker at 80 rpm at. Cultures were inoculated with a small piece of mycelium from working culture plates.

**Table 1:** Strains of *Stachybotrys* (*S*.) *chartarum* (*n* = 27) and *S. chlorohalonata* (*n* = 1) used during the current study, their assignment to genotype and chemotype using different methods, and their ability to produce macrocyclic trichothecenes

ID	Origin	Source	Genotype identification		MALDI-TOF MS identification	Macrocyclic trichothecenes^b^
			*tri5*-sequencing	Triplex PCR typing		
CBS 129.13^a^	Unknown	CBS	A	A	*S. chartarum*	–
S 1074	Indoor	LLS	A	A	*S. chartarum*	–
S 1244	Indoor	LLS	A	A	*S. chartarum*	–
S 1286	Indoor	LLS	A	A	*S. chartarum*	–
S 1348	Indoor	LLS	A	A	*S. chartarum*	–
S 1285	Indoor	LLS	A	H	*S. chartarum*	–
S 1335	Indoor	LLS	A	H	*S. chartarum*	–
S 1342	Indoor	LLS	A	H	*S. chartarum*	–
S 16St	Feed	LLS	S	S	*S. chartarum*	+
S 48St	Feed	LLS	S	S	*S. chartarum*	+
S 35It	Feed	LLS	S	S	*S. chartarum*	+
SBO1a^c^	Food	LLS	S	S	*S. chartarum*	+
SBO1b^c^	Food	LLS	S	S	*S. chartarum*	+
SBO2^c^	Food	LLS	S	S	*S. chartarum*	+
ATCC 34916^a^	Feed	ATCC	S	S	*S. chartarum*	+
IBT 40293^a^	Indoor	IBT	S	S	*S. chartarum*	+
S 1493/1	Indoor	LLS	S	S	*S. chartarum*	+
S 9	Indoor	LLS	S	S	*S. chartarum*	+
Sp 2675	Indoor	IBT	S	S	*S. chartarum*	+
S 1114	Indoor	LLS	S	S	*S. chartarum*	+
CBS 414.95^a^	Unknown	CBS	S	S	*S. chartarum*	+
CBS 324.65^a^	Indoor	CBS	S	H	*S. chartarum*	–
HMRB10	Indoor	IBT	S	H	*S. chartarum*	–
S 1339	Indoor	LLS	S	H	*S. chartarum*	–
S 1341	Indoor	LLS	S	H	*S. chartarum*	–
S 3	Indoor	LLS	S	H	*S. chartarum*	–
S 6	Feed	LLS	S	H	*S. chartarum*	–
CBS 413.95^a^	Unknown	CBS	*chlorohalonata*	*chlorohalonata*	*S. chlorohalonata*	–

*CBS* Westerdijk Fungal Biodiversity Institute, Utrecht, The Netherlands; *LLS* Chair of Food Safety, Ludwig-Maximilian-University, Munich, Germany; *IBT* Culture Collection of Fungi, Danish Technical University, Lyngby, Denmark; *ATCC* American Type Culture Collection, Manassas, USA; − not detected; + detected

^a^Reference strains

^b^Detected by LC-MS/MS: roridin E; L-2; verrucarin J; satratoxin F, G, and H

^c^Biermaier et al. (2015)

### Isolation of fungal DNA and PCR products

Fungal mycelia were vacuum-filtered through sterile 70-mm-diameter filter discs MN 615 (Macherey-Nagel, Düren, Germany) and washed twice with sterile tap water. Extraction of DNA from 100 mg mycelia was performed using the peqGOLD Fungal DNA Mini kit (VWR International, Erlangen, Germany) according to the manufacturer’s instructions. Extraction of DNA from agarose gels was performed using the MinElute Gel Extraction kit (QIAGEN, Hilden, Germany) according to the manufacturer’s instructions. Genomic DNA was eluted and suspended in filtered, sterile, deionized water for all extraction protocols (Filtropur S 0.2 μm, Sarstedt, Nümbrecht, Germany). DNA concentrations were determined with a NanoDrop 1000 spectrophotometer (PeQlab, Erlangen, Germany).

### Primer design

Primers for the detection of each *SAT* and *ATR* gene in *S. chartarum* and *S. chlorohalonata* were designed using sequence information available from the National Center for Biotechnology Information (NCBI, Bethesda, USA) database. Genome sequences of macrocyclic trichothecene-producing *S. chartarum* strains IBT 40293 (scaffold 155, accession KL650302.1) and IBT 7711 (scaffold 234, accession KL647604.1) as well as the atranone-producing strain IBT 40288 (scaffold 1, accession KL659150.1) and strain *S. chlorohalonata* IBT 40285 (scaffold 175, accession KL659308.1; Semeiks et al. 2014) were used to extract 21 *SAT* and 14 *ATR* gene sequences. Sequence alignments were made using the BioEdit software package version 7.2.5 (Hall 1999). The web-based tool “Primer Blast” (Ye et al. 2012) available at https://www.ncbi.nlm.nih.gov/tools/primer-blast/ was used for primer design with the following settings: product size 70–1000 bp, melting temperature 59–61 °C, automatic search mode, at least 5 mismatches to unintended targets, and maximum target size 4000 bp. All other settings were used in default mode. Differences in the individual product sizes allowed differentiation of fragments in a multiplex PCR assay. A list of the primers used in this study is given in Online Resource 1.

### DNA amplification and PCR product analysis

### Trichodiene synthase 5 (*tri5*) gene amplification and sequencing

The *tri5* PCR master mix contained per 50-μL reaction 5.0 μL of 10× *Taq* buffer, 5.0 μL MgCl_2_ (final concentration 0.025 mol/L), 5.0 μL dNTP (final concentration 0.002 mol/L), 12.5 μL *Taq* polymerase (Thermo Prime Taq DNA Polymerase, Thermo Scientific, Schwerte, Germany; final concentration 1.25 U/μL), 1 μL of each primer (tri5f/tri5r, final concentration 1 × 10^−5^ mol/L), and 50 μL sterile demineralized water. DNA amplification of the *TRI5* gene region was performed using the following temperature protocol: melting 1 × 95 °C for 10 min followed by 40× melting at 95 °C for 60 s, annealing at 51 °C for 30 s, elongation at 72 °C for 45 s followed by 1× final elongation at 72 °C for 10 min. The *TRI5* PCR product was sequenced to differentiate between *S. chartarum* type A and type S according to Cruse et al. (2002) and Andersen et al. (2003). Prior to sequencing, the *TRI5* PCR product was purified using the QIAquick® purification kit (Qiagen, Venlo, Germany) according to the manufacturer’s instructions and diluted with sterile demineralized water to the final concentration of 5 ng/μL. Sequencing was performed by Eurofins MWG Operon (Ebersberg, Germany). The obtained nucleotide sequences were compared with the National Center for Biotechnology Information (NCBI, http://www.ncbi.nlm.nih.gov/) and MycoBank (http://www.mycobank.org/quicksearch.aspx) (Robert et al. 2005) online databases using the BLAST tool (Altschul et al. 1990).

### PCR analysis of satratoxin and atranone cluster genes

Primers used in PCRs for the detection of the *SAT* and *ATR* genes are listed in Online Resource 1. Reactions were run as multiplex PCR using between three and five pairs of primers per reaction. Primers were selected to result in a common melting temperature of 60 °C and combined to result in products with clearly distinct fragment sizes. The master mix contained per 25-μL reaction 2.5 μL of 10× *Taq* buffer including 0.015 mol/L MgCl_2_ (MP Biomedicals, Heidelberg, Germany), 0.5 μL of dNTP solution (MP Biomedicals, 0.01 mol/L, A, T, G, C each), 0.5 μL of each primer (0.05 mol/L, custom synthesis by Eurofins, Ebersberg, Germany), 0.25 μL of *Taq* DNA polymerase (MP Biomedicals, 5 U/μL), and sterile demineralized water up to 24 μL. Formamide (0.25 μL per reaction) was added in the triplex PCR for the combined detection of the *S. chartarum* genotypes and *S. chlorohalonata*. One microliter of isolated genomic DNA (ca. 50 ng/μL) of the tester strains was added as template before the PCRs were run in a Master Cycler gradient 96 thermal cycler (Eppendorf, Hamburg, Germany) using the following temperature protocol: melting 1× at 94 °C for 5 min followed by 35× melting at 94 °C for 60 s, annealing at 59–62 °C for annealing time as given in Online Resource 1, elongation at 72 °C for 60 s followed by 1× final extension at 72 °C for 5 min. Elongation time during regular cycles was prolonged to 90 s for products exceeding 700 bp in size. Amplification products were separated on 1.3% (*w*/*v*) LE agarose (Biozym, Hessisch Oldendorf, Germany) prepared in 1× TAE buffer (0.05 mol/L Tris (pH 7.6), 0.02 mol/L acetic acid, 0.001 mol/L EDTA). The GeneRuler 100-bp ladder (Thermo Scientific, Schwerte, Germany) was used as molecular weight marker. Gels were stained with dimidium bromide (0.5 μg/mL) and inspected on a UVT 28M UV-transilluminator (Herolab, Wiesloch, Germany). Results were documented using a digital gel documentation system (Intas Science Imaging, Göttingen, Germany) and matching GDS frame grabber software (Intas, Göttingen, Germany).

### MALDI-TOF MS measurement

Mycelia of the analyzed strains were cultured and proteins were extracted according to Ulrich et al. (2016). MALDI-TOF MS measurements were performed using an Auto Flex Speed mass spectrometer (Bruker Daltoniks, Bremen, Germany) according to the Bruker Daltoniks guidelines (Bruker Daltoniks 2013). Each strain was measured in eight technical replicates, and data were processed using the Biotyper OC software (Bruker Daltoniks, Bremen, Germany). The Biotyper database (version 3.1.66) plus an inhouse database (Ulrich et al. 2016) was used for identification of strains to the species level.

### LC-MS/MS analysis—extraction method

For macrocyclic trichothecene analysis, each strain was cultured on three parallel MEA plates for 21 days at 25 °C and 95% air humidity. Cultures were stored at − 20 °C until extraction. Prior to extraction, each of the three parallel plates was separately transferred to a mixing bag and 50 mL ACN/H_2_O (84/16, v/v) was added. Bags were treated for 5 min in a bag mixer (BagMixer 400, InterScience, St Nom la Bretèche, France). The sample extracts were filtered through a paper filter, and an aliquot of 5 mL was evaporated to dryness under a gentle flow of nitrogen at + 50 °C. The residues were suspended in 1 mL ACN/H_2_O (30/70, v/v) using ultrasonication (5 min) and were subsequently filtered through a polyvinylidene fluoride (PVDF) syringe filter (0.45 μm, Berrytec, Grünwald, Germany) into 1.5-mL glass sample vials.

### LC-MS/MS analysis—measurement

The LC-MS/MS system consisted of an HPLC apparatus (Shimadzu LC-20AB, SIL-20AC HT, CTO-20AC, CBM-20A, Duisburg, Germany) and an API 4000 triple quadrupole mass spectrometer (Sciex, Darmstadt, Germany). The substance-specific parameters used for multiple-reaction-monitoring (MRM) measurement are listed in Online Resource 2. The following source parameters were applied: ion spray voltage (ESI+), 5500 V; temperature, 550 °C; nebulizer gas, 50 psi; heating gas, 60 psi; curtain gas, 30 psi; and collision gas (nitrogen), level 9. A Synergi™ Polar-RP (150 × 2 mm, 4 μm; Phenomenex, Aschaffenburg, Germany) was used as an analytical column protected by a guard column. The binary linear gradient consisted of eluent A (deionized water containing 5 mmol/L ammonium formate, 0.1% formic acid) and eluent B (LC-MS-grade methanol, 5 mmol/L ammonium formate, 0.1% formic acid) with a flow rate of 0.4 mL/min: 0 min 10% B, 10 min 40% B, and 26 min 100% B. The column was equilibrated at starting conditions for 6 min prior to each run. Analyst (Version 1.6.2) and MultiQuant software (Version 3.0.1), both provided by Sciex (Darmstadt, Germany), were used for data acquisition and processing.

## Results and discussion

In this study, we analyzed 27 strains of *S. chartarum* and one strain of *S. chlorohalonata*. Using MALDI-TOF MS, the respective species identifications were confirmed for all strains (Table 1). Since *S. chartarum* strains cannot be further differentiated into chemotypes by this method (Ulrich et al. 2016), the production of macrocyclic trichothecenes was analyzed to assign chemotypes to the respective strains. According to the LC-MS/MS data, 13 of the 27 *S. chartarum* strains produced macrocyclic trichothecenes, whereas *S. chlorohalonata* and 14 other *S. chartarum* strains did not (Table 1). Further analysis revealed that all strains with a thymidine (T) at nucleotide position 279 of the *TRI5* gene (*S. chartarum* type A) did not produce macrocyclic trichothecenes (Andersen et al. 2003). All strains that produced macrocyclic trichothecenes harbored a cytidine (C) at the same position (*S. chartarum* type S). However, the type S configuration in nucleotide position 279 was also found in six of the *S. chartarum* strains that produced no macrocyclic trichothecenes. The latter finding indicates that in contrast to findings by Andersen et al. (2003), the *TRI5* gene sequence does not seem to be a reliable marker for the differentiation of *S. chartarum* strains that produce macrocyclic trichothecenes from those that do not. From the results, it can be concluded that new and reliable genetic markers are needed for a more accurate differentiation between high- and low-cytotoxic strains in *S. chartarum*.

### Development of a triplex PCR assay for chemotype differentiation

In order to differentiate strains of *S. chartarum* type A from type S as well as from *S. chlorohalonata*, PCR primers were designed to hybridize with genes *SAT19*, *ATR6*, and *ATR4*, respectively, to allow for specific identification. The *SAT19* gene was selected because it was supposed to be unique in type S of *S. chartarum* (Semeiks et al. 2014). Moreover, it showed a very high degree of sequence homology between reference strains and included only one intron. It was therefore chosen for the design of diagnostic PCR primers for the *S. chartarum* type S. The *ATR6* gene was supposed to be unique in *S. chartarum* type A strains and had only weak homologies with the *ATR6* sequence in *S. chlorohalonata*. In the current study, it was therefore used for the design of diagnostic PCR primers for *S. chartarum* type A strains with no cross reaction expected to occur with *S. chlorohalonata*. The *ATR4* gene included regions of low homology between *S. chartarum* and *S. chlorohalonata*. One such region was used to design diagnostic PCR primers for *S. chlorohalonata*. The three primer pairs were combined in a triplex PCR resulting in three DNA fragments of different sizes for the different types and species (see Online Resource 3). *S. chartarum* type A was characterized by a 230-bp fragment, whereas *S. chartarum* type S showed a fragment of 346 bp. Amplification of *S. chlorohalonata* DNA resulted in two fragments of 544 bp and 230 bp, respectively. The latter result suggested that *S. chlorohalonata* possesses both an *ATR6* gene and an *ATR4* gene and that the designed primers hybridize to both of them. In order to omit the production of a 230-bp product, formamide was added to the PCR which resulted in a weaker band but did not completely prevent this cross reaction. However, since the combination of a 544-bp fragment with a 230-bp fragment only occurred in *S. chlorohalonata*, the primers are still useful to identify strains of that species. All in all, results showed that the species and types could be readily distinguished by applying the developed triplex PCR assay.

### Screening of *S. chartarum* strains with triplex PCR

**Fig. 1:**
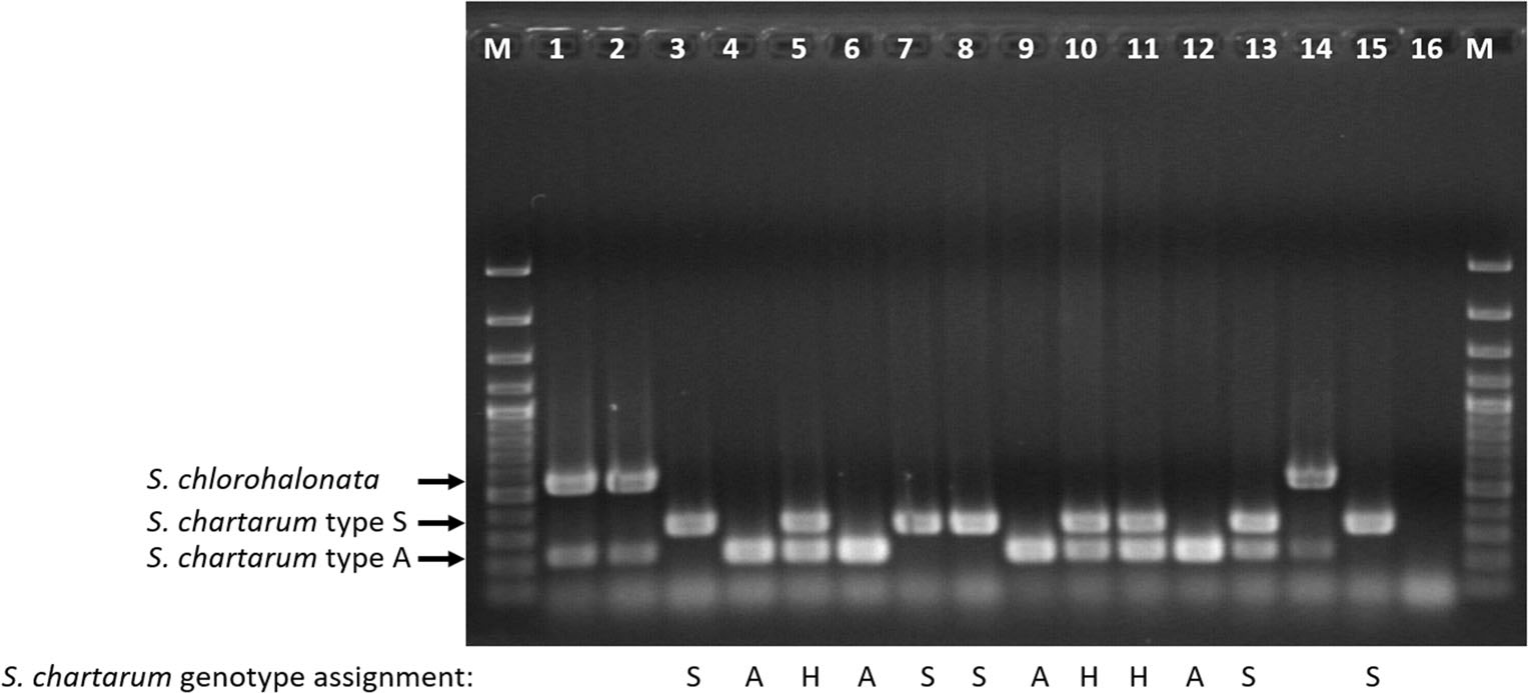
Triplex PCR for the combined identification of *Stachybotrys* (*S.*) *chartarum* type S and type A as well as *S. chlorohalonata*. Primers SAT19-Stype-f/r, ATR6-7Atype-f/r, and ATR4-1chloro-f/r were combined. Lanes: M = GeneRuler 100-bp ladder; lane 1 = *S*. *chlorohalonata* CBS 222.46; lane 2 = *S*. *chlorohalonata* CBS 329.37; lane 3 = *S. chartarum* Sp 2630; lane 4 = *S*. *chartarum* Sp 2674; lane 5 = *S. chartarum* Sp 2675; lane 6 = *S. chartarum* Sp 2676; lane 7 = *S. chartarum* 14/3; lane 8 = *S. chartarum* 95/121; lane 9 = *S. chartarum* D-9662; lane 10 = *S. chartarum* HMRF 4; lane 11 = *S. chartarum* HMRB 10; lane 12 = *S. chartarum* CBS 129.13; lane 13 = *S. chartarum* CBS 324.65; lane 14 = *S*. *chlorohalonata* CBS 413.95; lane 15 = *S. chartarum* CBS 414.95; lane 16 = no template control. Expected fragment lengths: *S. chartarum* S type 346 bp, *S. chartarum* A type 230 bp, *S. chlorohalonata* 544 bp + 230 bp. Different triplex PCR banding patterns are marked as genotype A (A), genotype S (S), and genotype H (H)

Applying the triplex PCR assay to a larger number of *S. chartarum* isolates revealed that not all isolates exhibited the expected banding pattern. Figure 1 displays 15 analyzed strains of which only 8 showed the banding pattern that was expected from the previously described results (see Online Resource 3). Four of the 15 *S. chartarum* strains produced fragments typical for type S (346 bp) together with fragments typical for type A (230 bp) (see Fig. 1, lanes 5, 10, 11, and 13). This result indicated that at least three distinct PCR patterns can be obtained from *S. chartarum* strains using the triplex PCR described above. Because the banding patterns were supposed to represent three different genetic constellations, three different genotypes were assigned to the corresponding patterns: genotype S (a single 346-bp fragment), genotype A (a single 230-bp fragment), and genotype H (a hybrid pattern with two fragments of 230 bp and 346 bp, respectively). Strains of *S. chlorohalonata* were characterized by two PCR amplicons of 544 bp and 230 bp as described above.

The characteristic amplicons of genotype H were also obtained in single-plex PCRs with the three different primer pairs (Online Resource 4) indicating that the distinct triplex PCR banding pattern of genotype H was not a result of primer interference. DNA was isolated from newly prepared single spore cultures of three *S. chartarum* genotype H strains as well as from one strain each of the *S. chartarum* genotypes S and A. The PCR analysis of samples resulted in the expected single amplicons for genotype S and A and confirmed the characteristic double band for genotype H (data not shown). The results confirmed that three separate banding patterns can be detected in strains of *S. chartarum* and that the double banding pattern observed in genotype H was no artifact from culture contamination. Therefore, the newly developed triplex PCR assay is a useful tool for the differentiation of *S. chartarum* S, A, and H genotypes.

### Genetic analysis of *S. chartarum* strains

**Fig. 2:**
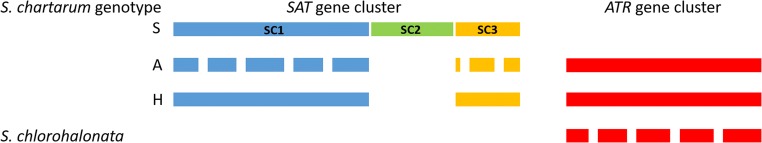
Schematic representation of the configuration of *SAT* genes and *ATR* genes in *S. chartarum* genotypes and in *S. chlorohalonata* according to PCR analysis. Blue, green, and orange boxes represent satratoxin core gene clusters SC1, SC22, and SC3, respectively. Red boxes represent the atranone core gene cluster. White areas represent missing genes

In order to further elucidate the genetic context for the triplex PCR assay, we analyzed the *SAT* and *ATR* gene clusters in 13 representative strains of *S. chartarum* and one strain of *S. chlorohalonata*. PCR primers were designed for the detection of each of the 21 genes in the *SAT* gene cluster and of 14 genes in the *ATR* gene cluster in all analyzed strains. Four strains each of *S. chartarum* genotypes S and H as well as five strains of genotype A and one strain of *S. chlorohalonata* were analyzed by PCR. Figure 2 shows a schematic representation of the configuration of *SAT*- and *ATR* genes in the different *S. chartarum* genotypes. Details on the presence (+) or absence (−) of each of the 21 *SAT* genes are depicted in Online Resource 5. The respective results for the 14 *ATR* genes are presented in Online Resource 6. Results showed that the four analyzed *S. chartarum* genotype S strains produced PCR signals for all 21 *SAT* genes. These strains were negative for all the 14 *ATR* genes (Online Resource 6). All five analyzed *S. chartarum* genotype A strains were PCR positive for all *ATR* genes (Online Resource 6). Three of these strains were negative for all *SAT* genes. However, strains S1286 and CBS 129.13 showed amplification products of the expected size for some but not all of the *SAT* genes. Neither of the two strains was PCR positive for any of the genes in the *SAT* core cluster 2 (SC2, genes *SAT11–SAT16*, according to Semeiks et al. 2014).

*S. chlorohalonata* CBS 413.95 was originally assigned to *S. chartarum* according to the reference culture collection. However, the strain had the banding pattern typical for the former species in the triplex PCR. The strain was PCR negative for all analyzed *SAT* genes indicating either that this gene cluster is absent in *S. chlorohalonata* or that genes are divergent from *S. chartarum*, so that it cannot be detected by our PCR assay. Regarding the atranone gene cluster, *S. chlorohalonata* CBS 413.95 was positive for eight of the 14 *ATR* genes (genes *ATR1*, *3*, *4*, *5*, *7*, *8*, *11*, *14*) but negative for six other *ATR* genes (*ATR2*, *6*, *9*, *10*, *12*, *13*). This finding suggests that some of the *ATR* genes were either missing in this species or genetically different from their homologs in *S. chartarum* genotype A and, therefore, do not allow amplification by PCR with the applied primers.

Strains of *S. chartarum* genotype H showed a distinct pattern of genes: all genes of the SC1 cluster (*SAT1*–*SAT10*) were detectable by PCR in the analyzed strains, whereas no PCR products were obtained for all six genes of the SC2 cluster (*SAT11*–*SAT16*). For the SC3 cluster (*SAT17*–*SAT21*), four of five genes were detectable in all strains. *SAT21*-specific PCR products were obtained for only two of the four genotype H strains. It was especially striking to see that the genotype H strains completely lacked the genes in the SC2 cluster and that this fact was correlated with their inability to produce macrocyclic trichothecenes (see Table 1). According to the data of this study, these mycotoxins were exclusively produced by *S. chartarum* genotype S strains, suggesting that the *SAT11–SAT16* genes are inevitable for the biosynthesis of macrocyclic trichothecenes. Moreover, they might be a useful genetic marker for high-cytotoxic strains of this species.

The genotype concept as developed during the current study is not fully congruent with the previously existing chemotype concept in *S. chartarum* because the new genotype H was not reflected as a type of its own. According to Desjardins (2008), independent parallel analysis using PCR-based techniques and chemical analysis of toxin production need to be performed to characterize a new genotype in mycotoxin-producing fungi. In the current study, analysis of the genetic configuration and ability to produce macrocyclic trichothecenes has revealed that the newly described genotype is unique among chemotype A strains of the species. Likewise, it is clearly genetically separate from chemotype A strains that harbor only the genes coding for atranone biosynthesis. During the current study, only a limited number of strains of the new genotype have been analyzed, both with respect to their genetic configuration and production of macrocyclic trichothecenes. It may therefore be possible that isolates exist in the field harboring some of the genes in SC2. Moreover, isolates may exist which can produce and excrete intermediates of the pathway for macrocyclic trichothecenes. Also, strains may exist that have replaced the function of missing genes by orthologues, as has been described in *Fusarium* spp. (Alexander et al. 2009). However, based on the data presented here, this is highly speculative and should therefore be further elucidated by analysis of a wider collection of isolates.

Data presented here cannot answer the question of the genetic origin of the new genotype. Three possible explanations for the observed genetic configuration in genotype H can be suggested, (i) genotype H has evolved independently from genotypes S and A, (ii) genotype H has evolved from genotype A strains that have picked up SC1 and SC3 by horizontal gene transfer, and (iii) genotype H has evolved from genotype S strains that have lost SC2 and picked up the complete ATR gene cluster by horizontal gene transfer. Such transfer of complete mycotoxin gene clusters was described for other mycotoxigenic fungi (Slot and Rokas 2011).

The existence of the new genotype H in *S. chartarum* challenges the concept of mutual exclusiveness of chemotype-specific gene clusters in *S. chartarum* (Semeiks et al. 2014). The results presented here show that also the differentiation of chemotypes, based on a nucleotide polymorphism in the *TRI5* gene of S*. chartarum*, does not fully correlate with the ability of strains to produce highly toxic satratoxins (Andersen et al. 2003). This observation is in agreement with studies by Peltola et al. (Peltolta et al. 2002) who developed a PCR assay based on the *TRI5* gene in *S. chartarum* and found that 40% of the PCR-positive isolates did not produce any macrocyclic trichothecenes. Since the PCR primers applied in this study were specific for type S strains, it can be speculated that the non-producers might have been strains of the new genotype H. Results obtained during the current study have shown that macrocyclic trichothecenes were only produced by strains identified as genotype S using the newly developed triplex PCR assay. It was demonstrated that the identification of strains that is based on sequence analysis of the *TRI5* gene does not differentiate properly between strains of genotypes S and H and will therefore overestimate the number of satratoxin producers among strains of *S. chartarum*.

It can be concluded here that the new triplex PCR assay provides a more reliable method for the identification of highly toxic *S. chartarum* strains. Moreover, the results obtained will be of great help in the development of DNA-based assays that can be useful as tools for the rapid and specific detection of satratoxin-producing *S. chartarum* strains in environmental samples.

### Funding information

Sponsored by the German Federal Ministry of Education and Research (BMBF) (grant no. 01PL17016) and the Brigitte and Wolfram Gedek Foundation, Ismaning, Germany.
